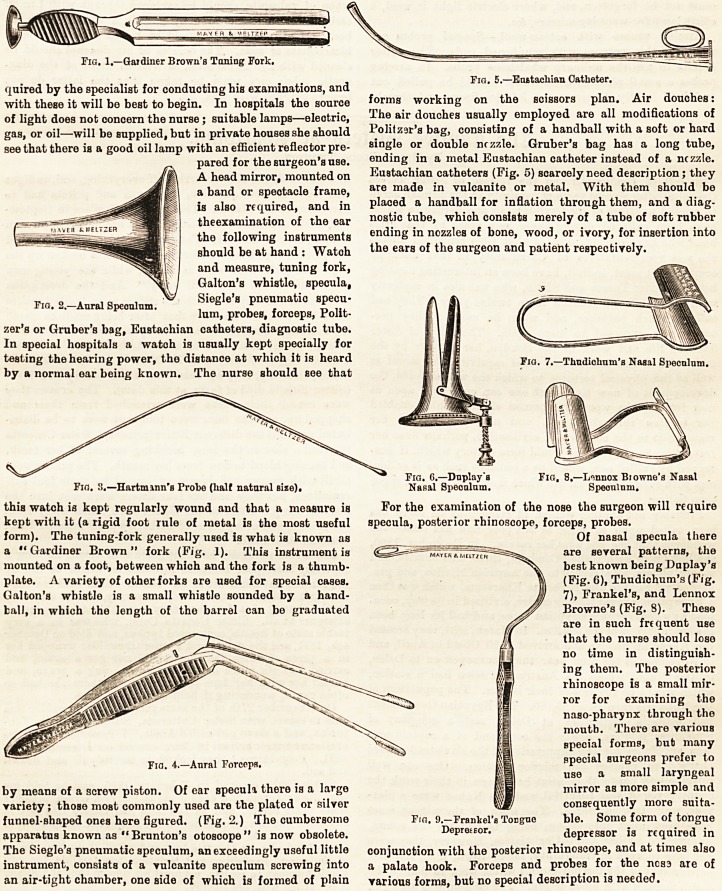# The Hospital. Nursing Mirror

**Published:** 1898-09-10

**Authors:** 


### "The Hospital" Nursing Mirror.

Being the Nursing Section of "The Hospital."

[Contributions for this Section of "The Hospital" should be addressed to the Editor, The Hospital, 28 & 29, Southampton Street, Strand, London, W.C., and should have the word "Nursing" plainly written in left-hand top corner of the envelope.]

### News from the Nursing World.

### THE NURSING SISTERS OF ST. JOHN THE DIVINE.

The Community of the Nursing Sisters of St. John the Divine is looking forward to the opening next month of the new building in Watson Street, Deptford, the construction of which is olmost finished. It is hoped that the Bishop of Rochester will perform the opening ceremony. His lordship is now visitor to the community, and his predecessors have been closely connected with the sisterhood in byegone days. The large and increasing work at Deptford is only one of the undertakings carried on by the sisterhood, other branches are St. John's Hospital, Lewisham, the Maternity Home at Gunter Grove, the Poplar District Home, and the Meyrick House Convalescent Home at Littlehampton. Part of an anonymous gift of £5,000 has been expended on the new house at Deptford, and the rest has been invested for the benefit of the work being carried on in the district. St. John's Hospital is also to be considerably enlarged, £3,000 and have been already given for the purpose.

### THE ABUSE OF STIMULANTS.

The abuse of stimulants is, alas, only too fatally easy to any whose work involves any mental strain, and this applies with special force to women. A very bad calamity has taken place in Calcutta from this cause. Mrs. Mary Niblet, nurse in the female ward at the General Hospital, was found intoxicated by the Acting Sister Superior, and, in consequence, reprimanded and ordered to her room. The unfortunate woman, believing that she would be dismissed, left the hospital, took a room at Hotel de Paris, and committed suicide by poisoning herself with prussic acid. Temperance is desirable in every walk of life, but to a nurse it is essential, and, whilst commiserating the fate of Nurse Niblet, it is only fair to her superiors to recognise the fact that they had no choice in the matter of administering a reprimand, and to extend to them our hearty sympathy.

### NURSE-TRAINING AT NORWICH.

The Norwich Guardians have attempted a new departure in the training of probationers, of which much that is favourable can be said. Considerable modifications of the scheme may be necessary before it can be put into practice, but they ought not to be impracticable. The Guardians approached the authorities of the Norfolk and Norwich Hospital, with a request that their probationers might be allowed to attend the lectures at the hospital, to sit for the examinations held at the conclusion, and to receive the certificate of having done so. This proposal amounts to the probationers receiving theoretical instruction at the hospital, and practical training at the workhouse infirmary, the Guardians bearing the whole of the expense. The hospital authorities, whilst willing to admit the probationers to lectures, will not give them examinations nor certificates. Nor can they be expected to give certificates to other than their own probationers. If, however, the Local Government Board can be prevailed upon to recognise a certificate under the above conditions, it is not improbable the Guardians could obtain its sanction to the appointment of an hon. board of examiners by the Guardians, which at the end of the three years' practical training at the workhouse hospital, combined with the theoretical instruction given at the hospital, would be able to grant the desired certificate. That is, of course, if the superintendent nurse at the workhouse infirmary were fully competent to teach.

### FEVER NURSING IN SUTHERLAND.

The County Council of Sutherland have voted an annual grant to the Sutherland Benefit Nursing Association on condition that one trained nurse be at the disposal of the medical officer of health for the county for cases of infectious disease. The Local Government Board have sanctioned the arrangement as an experiment, and call for a report on its working after a year's trial. "Should the present experiment give satisfaction, the Board will be prepared to consider a further scheme by the County Council on a larger basis."

### NURSING AT THE GENERAL HOSPITAL, MADRAS.

Considerable difficulty is experienced by the matron of the General Hospital, Madras, in getting the right kind of women for nurses. She and the surgeon think that this is because the prospects offered are not sufficiently good, but the Government attributes it to the fact that the Eurasian and East Indian classes consider the labour derogatory. The accommodation for the staff is also inadequate—a defect that will probably be rectified when the military authorities evacuate the Station Hospital, as is arranged, and the two hospitals are amalgamated. The Government, bearing in mind reports received two years ago, considers it necessary that the matron or assistant matron should always be on the premises at night, as it was found that neither the medical subordinates nor the senior Eurasian nurses could be relied upon to report properly neglect of duty by the nurses and attendants. One of our correspondents, in a letter from a plague hospital, regretted the fact that a young Hindoo widow who was helping in the wards had not the opportunity of being trained as a nurse; she showed a great aptitude for the work, and from these two instances it would seem that whilst some native women make excellent nurses, few of them are capable of organising and controlling subordinates, which is the chief duty of nurses occupying the higher positions.

### A NURSES' HOLIDAY.

The nurses employed at the Bradford Royal Infirmary, and the ten belonging to the Ladies' Guild for Nursing the Sick Poor, through the hospitality of Mr. Ree (a member of the board of management) and his wife, spent a delightful day at Scarborough. For the convenience of the infirmary management the nurses were divided into two parties, and the excursions took place on August 23rd and 30th respectively. On arrival breakfast was ready at the Balmoral Hotel, a substantial dinner was ordered for five o'clock, and tea was provided in the Spa Grounds, for which and for other entertainments the hosts had kindly procured tickets. Several members of the board of management and of the hon. medical staff availed themselves of Mr. and Mrs. Ree's invitation, and accompanied either one or other of the excursions. Needless to say, the guests enjoyed themselves thoroughly, and gratefully appreciated the kindness that had so thoughtfully provided for their pleasure in every way.

### POOR LAW INFIRMARIES AS TRAINING SCHOOLS.

There is no better field for training nurses for Poor Law and other institutions than the well-managed workhouse infirmaries and sick wards. If, therefore, the Guardians secure the services of capable and energetic superintendents, and make arrangements with their resident medical officers to supplement their work by lectures and other necessary teaching, they will have taken an important step forward in solving the problem of the deficient nurse supply. Two points are worthy of their attention, however, namely, that they cannot secure good officers unless the pay be adequate, nor will they attract the best kind of probationer unless suitable arrangements be made for their accommodation and comfort. The number of suitable candidates is limited (though there are unsuitable ones by the score), and the right kind of woman to make a good nurse is one who, not only appreciates, but requires provision for her own personal cleanliness and hygiene. It will not do, therefore, to crowd them in their sleeping apartments, and to serve their meals in a rough-and-ready fashion. Attention to these things is worth more than several pounds a year being added to the salaries. After all, one has only one life to live, and why should three years of it be made unhappy for want of good management? Besides, as environment is the most powerful factor in education, and slovenly habits once acquired are uncommonly hard to get rid of, why should it be neglected? There are ways by which the extra expenses thus incurred might be counteracted. In one workhouse no less than £100 was saved in one year by serving the bread ready cut on plates and allowing the inmates to help themselves to as much as they wanted instead of weighing out each his own portion.

### ROMSEY NURSES' HOME.

We understand that a freehold site for the Romsey Nurses' Home has been acquired in Cherville Street, at a cost of £150. As soon as the Corporation has approved of the plans the work will be begun. There will be two wards in addition to the nurses' accommodation, one for the reception of accidents and the other for cases of non-infectious sickness. The total outlay is estimated at £577, of which sum £517 has been subscribed.

### GOOD NEWS FOR A NURSE.

The present matron of the Northwich Infectious Hospital is Mrs. Neufeld, a native of Cheshire. Eleven years ago her husband, Mr. Charles Neufeld, was taken prisoner by the Khalifa, in whose camp he has been a captive ever since. One result of the splendid success of the Sirdar is to set him free, so that one of our nurses will have personal reasons for rejoicing.

### HOUNSLOW JUBILEE MEMORIAL.

A creche has been built at Hounslow by Mr. and Mrs. Ellis in memory of their son, and also as a Jubilee memorial. It consists of a large room for the children, 24 ft. by 18 ft., a small bedroom, kitchen, bath, and committee-rooms on the ground floor, whilst the matron's residence and servants' accommodation is provided upstairs. The outside appearance is good and picturesque. Mr. H. Ward, of Hastings, is the architect, and Mr. Billins, of Hounslow, is the builder. The crèche is much needed in the neighbourhood.

### SHORT ITEMS.

On the 24th inst., at St. George's Church, Jesmond, Newcastle-on-Tyne, Miss Rimington, until recently Lady Superintendent of the Ingham Infirmary, South Shields, was married to Mr. Henry Hunter, M.R.C.V.S. —The Liversedge Nursing Association, which was founded about a year ago, has received a donation for permanent investment of £500, from Mr. J. S. Cooke. The money will be invested in the names of the Chairman and members of the District Council as trustees. —The Rev. W. Hopkinson has acknowledged the receipt of £2 6s. on behalf of the Castor District Nurses' Fund; the result of the band parade through the village.—The annual report of the Honiton Medical Officers' Nursing Association is satisfactory, in spite of a small deficit. The nurse has paid about 5,000 visits, and several influential residents in the neighbourhood have promised subscriptions.—Dr. J. C. Williams has been appointed hon. physician of the Church Army's Mission Nurses' Training Home in Nutford Place, W.—Lady Hermione Blackwood, daughter of the Marquis of Dufferin, is training as a nurse in Chelsea Infirmary.—Mr. Rogers, a member of the Wandsworth Poor Law Board, is heading a movement to reduce the hours on duty of the workhouse nurses of Clapham and Wandsworth to eight hours a day. Their hours at present are from seven in the morning to seven in the evening, with an allowance of one and a half hours for meals.—The first nurse, appointed to the Knockando Nursing Association which was founded as a memorial of the Diamond Jubilee, was compelled to resign from ill-health. Her successor is satisfactory, but, as a midwife is needed, the association has decided to send her to Edinburgh for training. An arrangement will be made that she must refund the money for her fees if she leaves before the expiration of two years.—The committee of the Aberlour Nursing Association announced, at the seventh annual meeting of its supporters, that an engagement had been made to fill the nurse's place whilst she was absent qualifying as midwife.—A two days' bazaar was opened on the 1st inst. at Tain, N.B., by Lady Mackenzie of Gairloch in aid of the nursing association and the library of the Mechanics' Institute. —The Hostel of St. Luke, 16, Nottingham Place, W., a nursing home for the clergy, is now reopened for the reception of patients. It had been closed for cleaning and painting.—A pretty and successful sale of work was held lately at the residence of Dr. Hime in aid of the Bunerana District Nursing Society at Clauin Fois.—Owing to ill-health Miss A. E. Briggs has had to leave the Army Nursing Service.

### Nursing in Diseases of the Throat, Nose, and Ear.

By MACLEOD YEARSLEY, F.R.C.S.Eng., Assistant Surgeon to the Royal Ear Hospital, Surgeon-in-Charge of the Department for Diseases of the Throat, Nose, and Ear, the Farringdon General Dispensary, Hon. Surgeon for Diseases of the Throat and Ear, the Governesses' Home, &c.

### II.—GENERAL INSTRUCTIONS.

IN treating diseases of the throat, nose, and ear there are numerous small duties which the nurse may at any time be called upon to perform, and upon which the efficacy of the treatment will largely depend. Besides these manipulations every nurse should be acquainted with the instruments re-

FIG. 1.—Gardiner Brown's Tuning Fork.

quired by the specialist for conducting his examinations, and with these it will be best to begin. In hospitals the source of light does not concern the nurse; suitable lamps—electric, gas, or oil—will be supplied, but in private houses she should see that there is a good oil lamp with an efficient reflector prepared for the surgeon's use. A head mirror, mounted on a band or spectacle frame, is also required, and in theexamination of the ear the following instruments should be at hand: Watch and measure, tuning fork, Galton's whistle, specula, Siegle's pneumatic speculum, probes, forceps, Politzer's or Gruber's bag, Eustachian catheters, diagnostic tube. In special hospitals a watch is usually kept specially for testing the hearing power, the distance at which it is heard by a normal ear being known. The nurse should see that

FIG. 2.—Aural Speculum.

FIG. 3.—Hartmann's Probe (half natural size).

this watch is kept regularly wound and that a measure is kept with it (a rigid foot rule of metal is the most useful form). The tuning-fork generally used is what is known as a "Gardiner Brown" fork (Fig. 1). This instrument is mounted on a foot, between which and the fork is a thumbplate. A variety of other forks are used for special cases. Galton's whistle is a small whistle sounded by a handball, in which the length of the barrel can be graduated

FIG. 4.—Aural Forceps.

by means of a screw piston. Of ear specula there is a large variety; those most commonly used are the plated or silver funnel-shaped ones here figured. (Fig. 2.) The cumbersome apparatus known as "Brunton's otoscope" is now obsolete. The Siegle's pneumatic speculum, an exceedingly useful little instrument, consists of a vulcanite speculum screwing into an air-tight chamber, one side of which is formed of plain glass or a lens; from the chamber leads a tube ending in a mouthpiece or, better, a small hand-ball. Ear probes are of various shapes; a valuable form is Hartmann's. (Fig. 3) Blunt-pointed whalebone probes are very useful as cotton-wool holders. Forceps are, again, of varying shapes, from simple angular bow forceps (Fig. 4) to more complicated

FIG. 5.—Eustachian Catheter.

forms working on the scissors plan. Air douches: The air douches usually employed are all modifications of Politzer's bag, consisting of a handball with a soft or hard single or double nozzle. Gruber's bag has a long tube, ending in a metal Eustachian catheter instead of a nozzle. Eustachian catheters (Fig. 5) scarcely need description; they are made in vulcanite or metal. With them should be placed a handball for inflation through them, and a diagnostic tube, which consists merely of a tube of soft rubber ending in nozzles of bone, wood, or ivory, for insertion into the ears of the surgeon and patient respectively.

FIG. 6.—Duplay's Nasal Speculum.

FIG. 7.—Thudichum's Nasal Speculum.

FIG. 8.—Lennox Browne's Nasal Speculum.

For the examination of the nose the surgeon will require specula, posterior rhinoscope, forceps, probes.

Of nasal specula there are several patterns, the best known being Duplay's (Fig. 6), Thudichum's (Fig. 7), Frankel's, and Lennox Browne's (Fig. 8). These are in such frequent use that the nurse should lose no time in distinguishing them. The posterior rhinoscope is a small mirror for examining the naso-pharynx through the mouth. There are various special forms, but many special surgeons prefer to use a small laryngeal mirror as more simple and consequently more suitable. Some form of tongue depressor is required in conjunction with the posterior rhinoscope, and at times also a palate hook. Forceps and probes for the nose are of various forms, but no special description is needed.

FIG. 9.—Frankel's Tongue Depressor.

In examining the throat and larynx a tongue depressor, tongue cloths, and laryngeal mirrors are required. The most frequently used tongue depressor is Frankel's (Fig. 9). Laryngeal mirrors are too well known to need description. As tongue cloths, small squares of old linen, measuring about six inches by four, may be provided in sufficient number to allow of least one to each patient. The Japanese paper tongue holders used at some hospitals are useful, as they are very cheap and can be burnt after use.

A bowl of 1 in 40 carbolic or other antiseptic and a towel must not be forgotten, and, where electric light is used, a spirit lamp for warming mirrors, &c.

Arming probes with cotton-wool.—Special probes are made for this purpose with roughened ends. The writer prefers to use the ordinary whalebone probe. In arming probes a small piece of cotton-wool should be pulled out and held lightly between the left forefinger and thumb. The probe, held by the same digits of the right hand, is laid upon one corner of the wool, which, being steadied by the left thumb and forefinger, is wrapped round the end of the probe by a rolling motion of the latter. There is a certain "knack" in doing this properly. The wool requires wrapping round the probe evenly and with sufficient firmness to prevent its coming off during use, but not so tightly as to make it difficult to remove when soiled.

The nurse should take care to keep the instruments described above scrupulously clean. Eustachian catheters, when of vulcanite, should be syringed through with 1 in 20 carbolic lotion and cleared by passing a wire through the bore. If of metal they should be boiled in a solution of bicarbonate of soda. The nozzles of air douches should be cleaned with 1 in 20 carbolic, as well as those of the diagnostic tube; care must be taken that the latter do not become clogged with wax, &c.

### The Captive Sisters at Khartoum.

By HELEN FOGGO THOMSON.

THE fall of Omdurman and the release of Sister Teresa Gregolini after sixteen years of captivity in the Soudan serve to remind us afresh of the terrible trials and hardships which have been suffered during these long years by that little band who have during most of that time remained almost the sole representatives of Christianity in that unhappy country. It must, indeed, have been an interesting meeting between Sister Teresa and Slatin, who was also in captivity with the Mahdi and Khalifa some twelve years. They had suffered much together, and when he escaped they probably little expected to meet again. The story of Sister Teresa's life and work in the Soudan, her capture by the emissaries of the Mahdi, her cruel captivity, the moral as well as the physical tortures to which she was exposed, the development of new ties which one can only look upon as new fetters, the wonderful human vitality which enabled her to live through it all, and now her release, her restoration to the comforts of civilisation, perhaps even her restoration to old friends, would form a story which, if portrayed by skilful pen, would be a romance such as is seldom met with even in that real life which is often so much stranger than fiction.

Probably not another woman of refinement on earth has been called upon to suffer as this poor nun has done—the wonder is that she has kept her reason. In December, 1880, she left Cairo with Bishop Comboni, three missionaries, and several sisters, all attached to the Austrian Mission, and proceeded viâ Suakin and Berber to Khartoum, which was then a large place, a great trading centre, civilised in its way, occupied by a considerable Egyptian force, and had for long been the headquarters of the mission. In March, 1881, they crossed the Kordofan deserts and arrived at El Obeid in April, and in November of the same year they journeyed on to Delen, in Dar Nuba, where the Austrian Mission had a station, which was to be the seat of their labour. The population of Dar Nuba was then about 50,000. The Egyptian Government had made a settlement at Delen, and a company of Soudanese soldiers, under the command of a captain who was appointed for the suppression of the slave trade, were charged to protect the mission station. One can well imagine how happy they must have been in their work the country round was beautiful, and the Nubas were a pleasant, well disposed people. The work of the mission must have been most interesting, being to educate the young, to nurse, to civilise, and to Christianise. However, they were not long to work in tranquility, for in April, 1882, the first murmurings of the terrible storms which were to deluge the entire Soudan with blood began. The mission was attacked and wrecked, the Nubas carried off everything, and, as flight was out of the question, the nuns and priests had to surrender to the Mahdi. This event took place in September, 1882, and since then Sister Teresa has never known an hour's peace or comfort. Father Ohrwalder, who was one of the priests, says that "After the mission was broken up the boys and girls were sold as slaves, while the young men were drafted into the Mahdi army." And the description he gives of the way in which the sisters were treated makes one's heart sore. He says that after being made to march from Delen barefooted and carrying loads over a hot, thorny, desert road, and often without food or water for a whole day, they arrived at El Obeid in an exhausted condition, suffering, too, by this time from scurvy and fever (Sister Eulelia died of fever at this date). The crosses they wore round their necks were wrenched from them and chopped up, and the nuns were told they were to be distributed amongst the different Emirs (princes). Sister Concetta received a blow in the face, breaking several of her teeth, and causing blood to flow from her mouth. The priests were all ill with scurvy and dysentery, caused by their foul surroundings, but they and the four sisters were sent into the Dervish camp. Later on the sisters were taken away from the priests, and every form of barbarity used towards them to shake their faith.

One would naturally have supposed that during so long a captivity these poor innocent missionaries would be fed by the Mahdi, but not a bit of it. Slatin Pasha told me they were left without clothes, food, or money, and it was only by the charity of a few that they were able to eke out an existence at all. Sister Conatta Corsi, who was in a very feeble state of health, contracted typhus, and died on October 4th, 1891, and the sisters and Father Ohrwalder wrapped her in a piece of linen, as they could not get a coffin, and carried her six miles out of the town, dug a grave, and buried her with her face towards home, where she had so often gazed, wondering if help would ever come.

On November 27th of the same year Father Ohrwalder was able to escape with Sister Catterena, Sister Elizabeth Venturina, and a slave girl called Adela. I remember well the excitement their arrival in Cairo caused on December 21st, 1891. Poor things! they looked so terribly ill and drawn and sad.

Slatin Pasha escaped in February, 1895, and he told me that Sister Teresa had been made to marry a Greek prisoner some years before, and as she thus had family ties it was advisable not to approach her on the subject of escape, which in her case would be impossible. She was the only sister left behind alive in the Soudan, and after these long years of suffering, hardship, and isolation too terrible for any Englishwoman to realise, we must all rejoice that at last she is rewarded with her liberty.

### Training in the Provinces.

(*Continued from page* 188.)

### EDINBURGH ROYAL INFIRMARY (740 Beds).

### Terms of Training.

Three years' training is offered to women between 25 and 35 years of age at the Edinburgh Royal Infirmary. No certificate is given until the satisfactory completion of this term, but the regulations state that "at the close of a year their training as probationers will usually be considered complete, and during the two years next succeeding they will be required to enter into service on the staff of the infirmary." During the first year probationers are paid £10, for the second they receive £20, and for the third £21, with laundry and indoor uniform. Nurses who have satisfactorily finished their first year's training usually serve during the second as night nurses in charge of wards, or for special or extra duty. All nurses who have completed their full three years, and received a certificate, are eligible for promotion to the post of head nurse, and are so promoted in accordance with their fitness for the vacancies that may occur. The salaries of nurses on the staff range from £20, rising £1 a year to £25. Head nurses' salaries begin at £30, increasing by £2 per annum to £40. A very limited number of "special" or paying probationers are received for a less term than three years, on the following conditions: The pupil will, instead of receiving salary, pay the infirmary for the first quarter twelve guineas, for the second, eight; and for the third, five guineas; these sums to be paid in advance. The fourth quarter is free. The infirmary provides board, lodging, and uniform, with laundress. Probationers coming on these terms are subject to the same authority and have the same hours, duties, and other conditions as the other probationers. Certificates of efficiency are not given to those who do not undergo the full three years' training.

### Hours On and Off Duty.

Probationers' hours on duty in the wards range from 7 a.m. to 8.30 p.m., with time allowed for meals, and two hours daily for recreation. Half a day off duty is allowed every fortnight, and three weeks' holiday in the year. Head nurses are on duty from 7.30 a.m. to 8.30 p.m., with meal times, and two hours daily for recreation deducted from these hours. They have one day and one half-day off duty each month, and five weeks' annual leave. Assistant nurses' daily hours are the same as the probationers, but they take their two hours off in the afternoon instead of the morning, and have one day and one half-day's leave each month. Night nurses are on duty from 8 30 p.m. to 8 a.m. Their recreation hours are from 9 to 11.30, and they have one night off duty each month.

On Sunday all nurses are off duty in rotation, either from 10 to 1.30, 2 to 5 p.m., or 5.30 to 9.30. All are expected to "attend Divine service" once on Sundays.

### Meals.

The hours for meals are as follows: Breakfast, 6 30 a.m.; lunch, 9.15 to 10 a m.; dinner, 2.30 or 3 p.m.; tea, 4.30 to 6 p.m.; and supper, 8.45 p.m. The head nurses have all their meals, except dinner, served in their own sitting-rooms. All the nurses' meals are served in the dining-room. A certain proportion of "stores" are given out to the nurses weekly, and these are kept in the dining-room, each nurse having a locked cupboard for her special use.

### ROYAL HOSPITAL FOR SICK CHILDREN, EDINBURGH (120 Beds).

### Terms of Training.

Three years' training is offered at the Edinburgh Royal Hospital for Sick Children to candidates between the ages of 22 and 30, a certificate being granted on satisfactorily completing the engagement and passing examination. Lectures are given to the nurses on hygiene, physiology, medical and surgical nursing, by members of the medical staff and the matron. No salary is paid for the first year, beginning at £12 the second year, and increasing the third year to £16. Staff nurses receive £24 per annum, and sisters £30. Laundry and indoor and outdoor uniform are provided by the hospital.

Three years' full general training is an essential qualification for appointment as sister, candidates being required to hold a certificate for this and to have acted as staff nurse for at least a year. Preference is given to those having had children's training. Probationers in their third year are made staff nurses if they are suitable.

Night duty is taken by the first and second year probationers, in alternate periods of three months, the proportion being two nurses in each ward of 24 beds and one night sister.

### Hours On and Off Duty.

Probationers are on duty in the wards between 7 a.m. and 8.10 p.m., from which must be deducted time for meals and two hours off duty daily, from 2 to 4 or 4.30 to 6.30 p.m. On Sundays they are off duty from 9 a.m. to 12.50 p.m., 2 to 5 30 p.m., or 5.30 to 9.30 p.m. One whole day off is allowed once a month, on which the nurses do not enter the wards at all, being off duty from 8 p.m. the previous evening. For annual holiday probationers have one week's leave at the end of their first six months and 16 days in the summer; in the second year and after, three weeks and two days. At the end of three months' night duty nurses have four nights and three days off duty.

The staff nurses' daily hours on duty are similar to the probationers'; they go off duty each day alternately with the sister from 2 to 3 p.m. or 3 to 6 30 p.m. Once a fortnight they have leave from Saturday, at 3 p.m., to Sunday, at 9.30 p.m., with three weeks and two days' annual holiday. The sisters are on duty from 7.30 a.m. to 8.40 p.m., going off duty in the afternoon alternatively with their staff nurses for the same hours. Once a fortnight they have leave from Saturday, at 3 p.m., to Monday, at 9 a.m., with one month and two days' annual holiday.

### Meals.

The hours of meals for the nurses are as follows: Breakfast, 6.30 a.m.; lunch, 9 to 9.50 a.m.; dinner, 1 to 1.30 p.m., or 1.30 to 2 p.m.; tea, 4 to 5 p.m.; supper, 8.20 p.m. The sisters breakfast at 7; lunch is at 1 to 2 p.m.; tea, 4 to 5 p.m.; and dinner at 6.30 p.m. Night nurses breakfast at 8 p.m., and dine at 8 a.m. All meals are served in the dining-room, with the exception of the night nurses' midnight meal and their early morning porridge, which are eaten in the ward kitchens. A certain proportion of the night nurses' stores for these meals is sent to their special cupboards in the ward or ward kitchen by the ward maids.

### The Life of an Incurable.

The marvellous tenacity of life shown by some people has just been illustrated by the recent death of Miss Martha Cox, who for the last thirty-seven years has been a pensioner of the British Home for Incurables. During that period she has received £740—a large sum, but one that surely has been well spent. It is not a little thing to make the life of one physically afflicted comfortable for so long a time.

### Wants and Workers.

Nurse Hamblin, 53, Cadogan Street, Chelsea, S.W., has for disposal a silk elastic anklet, measuring E., 7 in.; N., 7¾ in.; T., 6¾ in. Price 3s. 6d.

### The American Red Cross Society.

### LETTER FROM THE SECRETARY.

THE following is a letter from Mrs. Whitelaw Reid, dated August 6th, 1898, to the Editor of the *New York Herald*:—

"I have failed to acknowledge personally to you until now the very generous cheques received by our society from the fund started by you, because, having been chairman of the committee for placing trained nurses, as well as secretary for our auxiliary, I have really had more to do than I knew how to accomplish; and with it all, our understanding with the Government was not satisfactory, so that Mrs. Cowdin and I had to go to Washington. We saw the President twice and also the Secretary of War and the surgeon-generals of the army and navy. The result of these interviews was entirely satisfactory, the Government agreeing to use our nurses in every possible way in their general and division hospitals and in the camps, and in consequence we have already sent nurses to the Sister Hospital, at Chickamauga; to Fort Wadsworth, Tampa; Fortress Monroe, Marine Hospital, Long Island; Fort Hamilton, Portsmouth, Charleston, and forty nurses and surgeons on the steamer 'Sampasas' with General Miles, to Porto Rico. We are also about to supply ten men for the hospital ship 'Missouri,' and two hospital superintendents are to leave to-day or to morrow with twenty nurses to take charge of the field hospitals at Chickamauga.

"This last is the beginning only of very important work, made possible by the new arrangement with the Government, and we hope in time to supply all the great army encampments. We furnish beds, bedding, transportation and mess tents, for the nurses, and the Government pays their salaries, allowing eight or ten nurses and a superintendent to each division hospital.

"We also had nurses under Miss Barton at Santiago, and no doubt the papers have told you of the splendid work done by them. Each group of nurses has a suitable matron at their head, and we have been successful in this matter, one being the superintendent of the Presbyterian hospital in New York, another the superintendent of a Baltimore hospital, &c. Of course, the rule in the past for the Red Cross has been only to use volunteer service, but it does not seem to work well here. In Europe so many ladies give their time that it is no doubt different, but with the demand here for skilled labour we find it necessary to pay our nurses twenty-five dollars a month and so have the very best to be found. Transportation is also very heavy, the nurses sometimes being obliged to travel two nights and two days, so that you see we need a great deal of money, and when it is necessary to board them near the camp or hospital, it is quite expensive, as people take advantage of war prices, and many people will not take them on any terms, for fear of contagious fevers.

"These are the most serious things we have to combat at present, and if the poor soldiers cannot get good nurses, their lives cannot be saved. I have had a very interesting letter from our head nurse at Portsmouth, in which he speaks of the Spanish prisoners. He says there are a hundred and ninety sick in the charge of the six men from the Mill Training School. So you see how grateful we are for the most substantial aid rendered us by the Paris fund, and can feel assured that every dollar of it will be needed and as wisely expended as possible. Perhaps in Europe you hardly realise that while not many of our men have been wounded, the spread of fever is already appalling, and is rapidly increasing. At Chickamauga, where we had fifty thousand men encamped, many hundreds are ill with typhoid fever, and at Miami, Tampa, and Fort Alger the death-rate has been even worse, while at Santiago there are four thousand sick soldiers, with the number on the increase.

"Will you kindly express to our friends in Paris our gratitude for all they have done? I have written this long letter, thinking you might be interested in our work, and I can only add that your generous response to our need here for trained care for our sick soldiers has been most deeply appreciated, not only by our auxiliary, but by the public."

### Appointments.

ISOLATION HOSPITAL, STOCKTON-ON-TEES.—Miss I. F Matthewson has been appointed Matron of this hospital; she is at present on the staff of the City of Worcester Isolation Hospital, where her resignation has been received with general regret.

GLOUCESTER DISTRICT NURSING SOCIETY AND HOME.—Miss Emma Dudley was appointed Lady Superintendent of this society on September 5th. She is at present assistant superintendent at the Central Home of the Metropolitan Nursing Association, Bloomsbury Square.

ILFORD URBAN DISTRICT COUNCIL ISOLATION HOSPITAL, ILFORD.—On August 30th Miss Brown, who was trained at St. Bartholomew's Hospital, was appointed Matron here. She is at present matron of the Ilkeston Sanatorium.

### Minor Appointments.

CHICHESTER INFIRMARY.—Miss Florence Parkes informs us that she was trained at the Sheffield Royal Infirmary—not at the Sheffield Royal Hospital, as was stated; and that, after the completion of her training, she remained as staff nurse to the theatre and theatre wards. She was, previous to her first appointment at Chichester, sister-in-charge of the Aston District Nursing Association, Birmingham; and we recorded her reappointment last week to the theatre and men's wards at Chichester.

SWANSEA GENERAL AND EYE HOSPITAL—Miss M. Bridger has been appointed Night Superintendent of this institution. She was trained for three years at the Huddersfield General Infirmary, where she remained for some months after taking her certificate. She then became head nurse at the Derby Hospital for Women, where she occasionally acted as temporary matron, and she has been head nurse of the Wolverhampton General Infirmary for nearly two years.

ROYAL ALBERT EDWARD INFIRMARY AND DISPENSARY, WIGAN.—On September 2nd Miss Bertha Saville, who was trained at the Leicester Infirmary, was elected Sister at the above. She has previously acted as head nurse at Woolton Convalescent Hospital, and, for eighteen months, has held the appointment of night superintendent of the Salisbury Infirmary, where she has latterly taken matron's duty.

MONTAGU COTTAGE HOSPITAL, MEXBOROUGH, ROTHERHAM.—Miss A. G. Aldred was elected, on August 27th, out of fifty candidates, Matron of the above hospital. Her previous appointments have been staff nurse at Jaffray Hospital, near Birmingham; charge nurse at Dewsbury General Infirmary; at Hammerwich Cottage Hospital; at the Derbyshire Royal Infirmary; at St. George's Home, Sheffield; and she is now ward sister at Rotherham Hospital.

NEWTON ABBOT.—Miss Alice Petitt, who was elected on June 20th District Nurse of Newton Abbot, entered upon her duties on the 5th inst. She was trained by the Workhouse Nursing Association at Worcester General Infirmary, and afterwards became assistant nurse at Newton Abbot Workhouse Infirmary, and, later, charge nurse of the same institution.

ROYAL EYE HOSPITAL, SOUTHWARK, S.E.—Miss Christine Jensen was appointed Charge Nurse of this hospital on September 6th. She was trained at the Swansea General and Eye Hospital, and has been charge nurse at Johannesburg Hospital.

### Everybody's Opinion.

[Correspondence on all subjects is invited, but we cannot in any way be responsible for the opinions expressed by our correspondents. No communication can be entertained if the name and address of the correspondent is not given, as a guarantee of good faith but not necessarily for publication, or unless one side of the paper only is written on.]

### ST. MARY'S DAY NURSERY.

MISS L. K. GIVEN WILSON, B.Sc., Secretary, St. Mary's Day Nursery and Hospital for Sick Children, Plaistow, E., writes: As the recent statements concerning the Plaistow Maternity Charity are calculated to do great harm to this institution, I beg that you will allow me to state that "St. Mary's Day Nursery and Children's Hospital," Plaistow, has no connection with Sister Katherine's Nursing Home and Maternity Charity. The fact that the two institutions are situated in the same district is a constant source of confusion.

### LADIES *V.* GENTLEWOMEN.

"A DOCTOR'S SON" writes: It is with regret that I read the vindictive letter of "A Doctor's Daughter" *re* the above in last week's number of your paper. And I cannot help thinking that if the writer was "a true gentlewoman" she would never have penned it, especially the latter part; as a person of good birth and education would never dream of hurting the feelings of those beneath her in station by pointing out the existing difference. She terms herself "A Doctor's Daughter," but this does not make her one whit more "a gentlewoman," as many doctors have sprung from the ranks of the "*οἱ πολλοί*." It does seem a pity that such an important personage as "A Doctor's Daughter" should be compelled to choose a profession in which she has to come in contact with those who in her estimation are inferior to her. It strikes me very forcibly that she wrote her letter out of nothing but petty jealousy on her part towards one whom she looks upon as a member (to use her own phrase) of "the servant class" who happens to be superior to her in hospital. Such a haughty contemptible spirit as "Doctor's Daughter" possesses only deserves to be crushed. And I for one should be glad to see the day when it would be impossible to find such a person in the ward of any hospital. At all events the sooner she gets this high-flown and ridiculous idea out of her "narrow mind" the better for herself and for the hospital in general which has been so unfortunate as to procure her services.

"BLUE BLOOD" writes: I have been much interested in some discussions which have lately been published in your paper. I thoroughly agree with "Matron"; it must be truly annoying to find that when advertising for nurses, and requiring for her needs women of gentle birth, that her advertisements are only answered by women who are anything but gentle. "Brighouse," in an article of last week's, leads one to understand that "any woman who lives an honest, upright life is necessarily a gentlewoman or lady." I beg to differ; a lady is a gentlewoman—it is only recently that the term "lady" has been illegitimately and wrongly used. A woman who is of good family and has been well brought up—good education, &c.—has more refined tastes and ideas than a woman, however high her principles may be, who has had practically no education, save, perhaps, that of the Board school, and later on moves in a society which is anything but gentle. A woman may have good instincts, live an honest, upright life, but that does not certify that she is a lady. Could one say that because a cart horse is perfect and in every way thoroughbred, that he might be "converted into" or "trained to be" a racehorse. No, certainly not! Surely "Brighouse's" experience with ladies must be very limited, or she must have come in contact with very questionable ladies. But, returning to her experiences with women, how seldom are all these high-born qualities which she speaks of met with in the lower classes? They certainly are met with occasionally, but then, again, these high principles must be in a very elementary stage and undeveloped owing to lack of education. I see, in your paper of August 13th, that "Lear" maintains that any woman may be a gentlewoman if she likes—if she likes—just as though by paying a certain sum down she might be duly registered as such, being at liberty to behave and conduct herself as she (a very questionable person) thought fit. Let us be content, and strive to become good and noble women, and ever bear in mind that "it is He who hath made us and not we ourselves."

"A. G." writes: I wonder who has instilled such ideas as to the essentials of a gentlewoman into the mind of "A Doctor's Daughter." The nature expressed is scarcely that for which one would seek among truly nobly-born British women, and more especially British nurses—those whose aim should be, not the gratification of their own pleasures and desires, not criticising members of the profession and evidently keeping down as much as possible, by the influence of a strongly-expressed prejudice, the apparently lowly born, but working heart and soul, with loving self-denial, to enrich the profession by their individual labour in so honourable a service. I also wonder if "A Doctor's Daughter" has forgotten who first relieved, and taught others to relieve the pain and suffering of mankind. He was a working man, a "refined and gentle" carpenter. Will "A Doctor's Daughter" hold up her hands in horror? Will she exclaim, "How common! How degrading to the profession?" Will she deny that he was a "gentleman?" I am afraid she must have failed to meet with many womanly women in her experience, or such extreme views could not be entertained. She forgets also that there are many great-hearted, noble women, who, because of this strong prejudice of the "classes," are withheld from the field of labour to which they are often better suited than their more prosperous and therefore "ladylike" sisters. I gladly state that I have realised the dignity of labour, and to my mind the greatest essential in entering such a profession as "nursing" is a whole-hearted devotion to the work of relieving and sympathising (a woman's peculiar privilege) in the trials and hardships of suffering humanity, and how can they conscientiously do this who degrade our civilised state by crying down the masses, among which are often found those who are fitted for a high mission in life. The idea that a well-educated, refined girl should be excluded from this noble profession because even bordering on the lowly-born is worse than cruel, and those who uphold such ideas should play no part in such matters; how much less should they be allowed to offer opinion on the subject of fit candidature for the profession.

### Sholapore Plague Hospital.

THE lady superintendent of the above hospital, in writing to the *Manchester Courier*, thus describes how she found it: The wards were huts, composed of matting walls, fastened together with bamboos, roofs of grass, and earth floors; slits in the matting for windows, and most of the wards had no doors. The beds were made of a framework, and short legs of rough wood, laced across with rope from side to side. . . . . There were patients on the floor, patients in the beds, and, for want of space, the cots were so close together that it was quite impossible to get between them. The floors were strewn with food and dirty dressings and piles of filthy clothes belonging to the patients' friends. The floors were also covered with expectoration.

### For Reading to the Sick.

"LORD, I am oppressed : undertake for me."—*Is.* xxxviii. 14.

The day is over, the feverish careful day;
Can I recover strength that has ebbed away?
Can even sleep such freshness give, that I again
should wish to live?
Let me lie down! No more I seek to have
A Heavenly crown; give me a quiet grave;
Release and not reward, I ask—too hard for me
Life's daily task. —*T. T. Lynch.*

Not now, my child!—a little more rough tossing,
A little longer on the billows' foam,
A few more journeyings in the desert darkness—
And then the sunshine of thy Father's home!
Not now! for I have wandered in the distance,
And thou must call them in with patient love;
Not now! for I have sheep upon the mountains,
And thou must follow them where'er they rove.
Not now! for I have loved ones, sad and weary—
Wilt thou not cheer them with a kindly smile?
Sick ones who need thee in their lonely sorrow—
Wilt thou not tend them yet a little while?
—*C. P.*

Spirits! that round the sick man's bed
Watch'd, noting down each prayer he made,
Were your unerring roll displayed
The pride of health t'abase. . . .
How should we gaze in trance of fear! —*Keble.*

Pain, that to us mortal clings,
Is but the pushing of our wings,
That we have no use for yet;
And the uprooting of our feet
From the soil where they are set,
And the land we reckon sweet.

Father! that in the olive shade,
When the dark hour came on,
Did'st, with a breath of heavenly aid,
Strengthen Thy Son.
Oh! by the anguish of that night,
Send us down bless'd relief;
Or to the chasten'd let Thy might
Hallow this grief! —*J. Ingelow.*

**Reading.**

On a sick bed both mind and body are oftentimes alike oppressed. We cannot think, we cannot read, we cannot reason, we cannot pray. The mind is confused, the circulation languid, the body full of pain. There are tossings to and fro, and turnings from side to side. "Would God it were morning," we say in the darkness. "Would God it were evening," we say in the day. These are Nature's helpless cries when left alone. She weeps, she faints, she craves for sympathy. She is powerless. When the soul is taken into fellowship with God, when Christ has been revealed to her as the hope of glory, when life and immortality have been brought to life by the Gospel—then she sees beyond, and has a prayer for the emergency. It is often as much a sigh as a prayer that God would "undertake" for her—put underneath the "everlasting arms," bid the Spirit help her infirmities—hold up the Cross of Christ—tell of His dying love—unveil the many mansions—fit alike for life and death. If this is but

. . . the burden of a sigh,
The falling of a tear;
The upward glancing of an eye,
When none but God is near,

God hears, and will undertake for us, and "oppression" shall give place to that liberty wherewith He makes His children free.—*From "Short Sermons for Sick Rooms."*

### Notes and Queries.

*Temporary Home.*

(221) Can anyone recommend a home for two or three months for a girl of 17 who is suffering from curvature and disease of the spine? Is able to wait upon herself entirely; only wants rest and good air, seaside preferred. Terms must be moderate.—*E. J. T*, 42, *Petherton Road, Highbury, London.*

"E. J. T." would find "Homes and Hospitals for the Benefit of Gentlewomen," published by the Army and Navy Stores, 105, Victoria Street, Westminster, at 1s., useful.

*Home for Slight Invalid.*

(222) Can you tell me of a home or institution, near London, where an elderly maiden lady, suffering from chronic ulceration in her leg (which she is able to attend to herself), could be received? She has the furniture of one room, and a small annuity.—*Inquirer.*

Consult Burdett's "Hospitals and Charities." See also "Homes and Hospitals for the Benefit of Gentlewomen," 1s. from Army and Navy Stores, Victoria Street, Westminster.

*Permission to Operate.*

(223) Is it lawful for a surgeon belonging to a hospital to operate upon a person without his or her consent, or without the permission of that person's relatives or friends? If the doctor does operate without permission, what remedy has the person operated upon, supposing the operation to be contrary to his or her wishes?—*Enquirer.*

A hospital is not a prison; every patient has a perfect right to leave the institution, and undoubtedly anyone who should interfere to prevent a patient from so doing would be liable to an action at law. Every patient, then, remaining in a hospital, and accepting the treatment there provided, gives an implied consent to what is done. Every patient, however, has a perfect right to refuse to undergo any proposed treatment or any proposed operation, and if, notwithstanding such refusal, the medical officer either forces the patient to undergo it, or takes advantage of the fact that the patient is unconscious (whether from the action of an anæsthetic or from the natural course of the disease) to do what the patient has prohibited him from doing, that medical officer will expose himself to an action at law for any damage which may result from his proceedings. Whether an action would lie if no damage could be proved we are not prepared to say.

*Dates of Entrance.*

(224) Can you kindly tell me if, in large hospitals or infirmaries, probationers are received separately or at one certain period, and is September a likely month?—*M.*

Probationers are received as vacancies occur. Apply at once to the matron of the hospital you wish to enter, and, after she has accepted you, she will inform you when a vacancy occurs.

*A Question of Title:.*

(225) Has a nurse who has had four years' training in a children's hospital, one year as sister of a children's ward, one year as sister of a children's and women's ward, and two years' private nursing, any right to call herself a trained nurse?—*C. S.*

Certainly she is trained for nursing children's diseases; nevertheless, she would be debarred from many posts, because she does not hold a three years' certificate from a general hospital.

*Fever Probation.*

(226) I am a dormitory maid, and wish to enter a fever hospital as probationer. How can I get references?—*A. H.*

You should answer the advertisements for probationers that appear frequently in our columns. Ask the lady under whom you are serving for a reference.

*References.*

(227) I should appreciate the favour of a reply from you stating if you think ——— Home all right or not. My sister answered the advertisement appearing in your columns, and received a favourable reply.

Whilst our manager exercises great care in sifting the advertisements in order that none but genuine ones may be inserted, it is impossible for us to act as references to any person or institution not personally known to ourselves. Why not ask for references, and write to those given?

*Epileptics.*

(228) Will you kindly tell me the addresses of one or two homes for epileptics.—*A. B.*

The Epileptic Colony, Chalfont St. Peter's, Bucks; the Meath Home for Epileptics, Godalming; and the Home for Epileptics, Maghull, near Liverpool.

*Cookery.*

(229) Can you tell me of any lectures on sick-room cookery which are given in London other than those by the National School of Cookery?—*Rosaline.*

The Royal British Nurses' Association arranged courses of sick cookery last winter. Apply The Secretary, 17, Old Cavendish Street, W. There are various other agencies which do the same, and the Secretary, the Victoria (Commemoration) Club, Southampton Street, Strand, might be able to tell you of any course now beginning.

*Spinal Curvature.*

(230) "Horton."—There is no one method of treating spinal curvature which is exclusively employed at the Liverpool Southern Hospital.

**ANSWERS REQUESTED.**

*To Keep Mattresses.*

(231) May I ask your numerous readers who are matrons of hospitals how they manage to keep the hair mattresses in the wards in a sanitary condition? I find that, in spite of mackintoshes, accidents often happen.—*Matron.*

*Sponges and Soap.*

(232) C. F. would be glad to know how to prepare new sponges for surgical operations. Also how to make soft soap fit for household scrubbing, using the common fat obtained from the kitchen.

## Figures and Tables

**Fig. 1. Fig. 5. Fig. 2. Fig. 3. Fig. 4. Fig. 6. Fig. 7. Fig. 8. Fig. 9. f1:**